# The Importance of Commmunication with the Primary Care Team

**Published:** 1988

**Authors:** John James

**Affiliations:** Montpelier Health Centre and Bristol Children's Hospital


### The Importance of Communication with the Primary Health Care Team

**John James**

Montpelier Health Centre and Bristol Children's Hospital

### INTRODUCTION

The management of neoplasia in childhood is highly specialised and, increasingly, takes place in the setting of major hospital units. The improved outlook for children with cancer owes much to the expertise and dedication of those working in these units; sophisticated treatment protocols and shared experiences between major units ensure continued improvement. Nevertheless, the whole family is affected by the disease and all aspects of management must be considered; physical management cannot take place in isolation. The psychological and sociological effects of the disease are equally important. Support for the family is essential, both at home and in the hospital. Support must be available in the community and the Primary Health Care Team is ideally placed. Despite the improved outlook for childhood neoplasia, a third of affected children will need terminal care and their families will need long-term bereavement counselling and follow-up.

Communication is an accepted tenet of good medical care, yet communication between hospitals and the community is, unfortunately, often poor. By considering in turn the disease itself, the facilities offered by specialist units and those offered by the community, the importance of good communication and shared care will be emphasized. The experience of the Oncology Unit at Bristol Royal Hospital for Sick Children, in developing an "oncology information pack" for members of the Primary Health Team, will be presented.

### THE DISEASE

Neoplasia in childhood occurs rarely. One in six hundred children under the age of fifteen will develop the disease and a General Practitioner is unlikely to see more than one or two cases in his career. Two thirds of children may now expect to be cured. The diagnosis can be difficult to make and there are often apparent delays before the General Practitioner refers to the hospital specialist. Treatment is usually based in highly specialised major paediatric oncology units which may be some miles away from the child's home. As more is known about childhood cancers, treatment protocols become increasingly complex as does the management of side effects of the treatment. General Practitioners cannot be expected to have an up-to-date knowledge of this management. Probably the most significant advances in treatment have resulted from major units using standardised treatment protocols, and pooling and acting upon the results. Again, these physical aspects of management are clearly beyond the brief of General Practitioners.

### THE HOSPITAL UNIT

Major hospital units are staffed by highly specialised and trained staff. Many of their referrals may have been made from local hospitals and not via the patient's own General Practioner. The unit may be many miles away from the patient's home. Families may find themselves isolated and confused; accommodation can often be a serious problem. Specialist diagnostic facilities will be available through the laboratory services as well as specialised radiological facilities. Where appropriate, surgery, radiotherapy and administration of complex drug protocols will be provided. Many units will have a Social Worker attached as well as a Child Psychologist and other psychiatric services. Some units will have nursing staff who are able to see the patient at home.

Parents generally develop enormous confidence in the staff of these units and themselves become rapidly conversant with the nature of their child's disease and the complex treatment protocols. Nevertheless, increasing importance is laid on the child spending as little time in hospital as possible; much is known about the emotional problems associated with in-patient hospital stay. After an initial period in hospital while the diagnosis is being established, a child may spend very few days in hospital over the ensuing months and years. It is the experience of most units that parents will contact the hospital directly, in preference to contacting their own General Practitioner. There is no doubt that specialised units can provide the best physical care for a child but they are less likely to be able to supply continual family support, much of which would probably take place at home. The family will be under much stress, even if the disease responds well to treatment; should terminal care become necessary (and this is increasingly managed from home), hospital teams are limited in their ability to visit at home. Bereavement affects the whole family and parents, brothers and sisters will need long-term and, possibly, intensive support. This must clearly be beyond the scope of a major hospital unit.

### THE PRIMARY HEALTH CARE TEAM

The Primary Health Care Team comprises health workers working in the community and includes General Practitioners, Health Visitors, Practice Nurses, and District Nurses. They are usually based in a Health Centre or surgery. Additional workers would include Social Workers (sometimes attached to a Practice) and personnel representing other services, such as physiotherapy, night sitting, appliances and home teaching. The Primary Health Care Team workers are likely to know the family very well and can provide intensive input if necessary. As childhood cancers can be difficult to diagnose, confidence in the General Practitioner (and Practice Nurses) may have been lost, particularly if there appeared to be a delay in making the diagnosis. As parents become familiar with even complex details of cancer management, it may become apparent to them that they have a better knowledge than their own General Practitioner. Once the diagnosis has been established and treatment initiated, the family will spend less time at a major unit and should be able to make use of the Primary

Health Care Team. Support for the family is best given in the home or community setting. The Primary Health Care Team is best qualified to provide long-term support, particularly if the disease is to prove fatal. Properly informed and trained General Practitioners and nurses should be able to administer some drugs and monitor blood counts. They should be able to give general advice about management of day-to-day problems. Unless members of the Primary Health Care Team are knowledgeable about these, and understand the principles of physical management, their role will be much diminished and the family will suffer. Their role in the management of childhood cancers should not simply be limited to supporting the family when active treatment has failed.

**Introduction of the Paediatric Oncology Information Pack for General Practitioners**

In order to help members of the Primary Health Care Team become more involved in the management of childhood cancer, an Information Pack was developed. Consisting of twelve sheets of A4 paper, it is sent to General Practitioners once a diagnosis has been made, detailing the diagnosis and explaining the organisation of the unit. The appropriate treatment protocol is provided and side effects of the drugs are detailed. A section on radiotherapy is included. The management of common anticipated problems, such as fever, neutropaenia, chicken pox and measles is outlined. Emphasis is laid on the team approach which involves hospital staff and the Primary Health Care Team. It was decided that, as policy, General Practitioners would be contacted by 'phone as soon as the diagnosis had been made. From the outset, the General Practitioner is encouraged to become involved in the management and this is made clear in the Information Pack. A questionnaire was sent out to 79 General Practitioners who had received the Information Pack over an eighteen month period from April 1984 to November 1985. Sixty (76%) completed the questionnaire. A quarter were aware of the diagnosis within 24 hours of the child being admitted to hospital. Over 90% found all aspects of the Information Package helpful. All had read it and two thirds had shown it to their partners. Just under half the General Practitioners showed the Information Pack to their Health Visitors and District Nurses. All General Practitioners felt it had helped them in dealing with the affected families and communication between the hospital and the community was improved. An interesting finding was that the hospital staff involved in preparing the Information Pack became more aware of the problems of those working in the community away from a major unit.

### CONCLUSIONS

The management of childhood neoplasia should encompass the physical, social and psychological aspects of the illness. Continuity and shared care between hospital and community workers is essential. This requires sharing of knowledge and both hospital and community being aware of their respective skills and the facilities that are available. Communication between the two is essential if the affected family is to receive optimum care and support. Irrespective of the outcome, it is the Primary Health Care Team who will be dealing with the family long after involvement with the major hospital unit has ceased. Communication between hospital and community is essential from the moment the child enters hospital for the first time.

